# Overview of Treatment Options for Mild Traumatic Brain Injury: A Literature Review

**DOI:** 10.7759/cureus.59021

**Published:** 2024-04-25

**Authors:** Hemangi Patel, Sneha Polam, Roody Joseph

**Affiliations:** 1 Sports Medicine, Nova Southeastern University Dr. Kiran C. Patel College of Osteopathic Medicine, Fort Lauderdale, USA

**Keywords:** return to play, mild traumatic brain injury (mtbi), return to learn, rehabilitation, concussion

## Abstract

The incidence and prevalence of concussion, a type of mild traumatic brain injury (mTBI), have steadily increased among athletes, both students and professionals, across a wide variety of sports, including, but not limited to, swimming, tennis, football, and boxing. Recent data have demonstrated that sports are one of the leading causes of concussions among student athletes. While the exact mechanism of concussion onset has yet to be fully elucidated, data suggest that the pathophysiology involves rotational acceleration and deceleration of the brain, leading to axon tearing and disturbance in the metabolic cascade of glucose. Concussive events can have debilitating effects on an athlete, including chronic traumatic encephalopathy (gradual degeneration of brain tissue) that is related to personality changes, emotional disorders, and even dementia. Common symptoms associated with concussion include dizziness, nausea, vomiting, and headaches. The physical assessment consists of a combination of tools involving the mental status examination, vital signs, cervical spine exam, eye exam, and neurological testing. The use of osteopathic manipulative medicine (OMM), pharmacotherapy, hyperbaric oxygen therapy (HBOT), aerobic exercise, balance, and/or vestibular therapy are many common treatment approaches for concussion and post-concussion sequelae. This literature review aims to provide insight into concussions, the current treatment options available, and the new developments in concussions per the Amsterdam 2022 International Consensus Statement on Concussion in Sport published in 2023.

## Introduction and background

A concussion is a type of mild traumatic brain injury (mTBI) that involves an insult to the head, with or without loss of consciousness, which can lead to long-term complications. While the exact mechanism of concussions needs to be fully elucidated, data suggest that the pathophysiology involves rotational acceleration and deceleration of the brain, leading to axon tearing and disturbance in the metabolic cascade of glucose [1]. The common symptoms of concussions typically include dizziness, nausea, vomiting, and headaches [2]. In athletics, the risk for concussion varies according to the type of sport, with some sports demonstrating a higher prevalence [2]. Specifically, collision sports such as boxing or football are associated with a higher incidence of acute brain injuries compared to non-contact sports like swimming or track [3]. Growing public attention has resulted in greater awareness of sports-related concussions. Furthermore, the media has been increasingly highlighting the severity of traumatic brain injuries in sports, specifically concussions [4].

As a result of the growing public concern and media coverage surrounding sports-related concussions, it is important to understand how these injuries are diagnosed and managed. The presence of a concussion is a medical diagnosis. Presently, there is no specific lab test or imaging that can be used for diagnosis. The duration of concussion symptoms is variable and can be dependent on several factors, such as age, severity of injury, injury history, and associated symptoms. Symptoms can range from short-term (days to weeks) to long-term (years). However, most concussion symptoms may resolve in days to weeks [4].

Furthermore, there is no definitive treatment approach for concussions. The recovery strategy currently used involves a graduated stepwise approach, beginning with a short period of 24-48 hours of rest [5]. It is recommended to remove the patient from any active sports participation for rest and allow them to be evaluated by a physician. Proper rest limits cognitive, physical, and social activities, which can lead to a reduction of symptoms, expediting the recovery time necessary [5]. Additionally, it prevents the possibility of a second concussion from occurring, which is important as it would worsen the outcome if a player were to be injured again. After rest and recovery, many other treatment approaches can be used. The Jugular vein compression (JVC) collar is an approach that helps to protect the brain during collisions in sports. Osteopathic manipulative medicine (OMM) helps to release tension in the cervical muscles. Herbal supplements and pharmacotherapy can assist in improving cognition, attention, and neuronal damage. Other treatment options include aerobic exercise, hyperbaric O_2_ therapy, blood flow restriction, and vestibular rehabilitation therapy to improve mobility [6-13].

Generally, the six-step return-to-play protocol provides a structured framework for safely reintegrating athletes into their sports following a concussion [5]. Initially, athletes must undergo a period of rest from both physical and cognitive activities. Following this, the second step introduces light aerobic exercise, such as walking or swimming. The third phase involves sport-specific exercises, such as running drills, but strictly prohibits any activities that may cause head impact. The fourth step includes more intense non-contact training drills, simulating sports demands without risk of collision [5]. Before advancing to the next step, a physician conducts a reassessment of the patient, ensuring they are medically cleared to proceed. The fifth step escalates to full-contact practice with normal training activities to gauge the athlete’s readiness for competition. The last step is a full return to play, contingent on the resolution of all concussion symptoms at each preceding stage. Additionally, a period of at least 24 hours between each step must be followed to monitor for symptom recurrence [5].

If symptoms of concussion persist for weeks to months, the athlete is likely experiencing post-concussion syndrome and should not return to play. Permanent removal from collision sports is advised if post-concussion syndrome persists for several months and the athlete experiences permanent neurologic deficits, movement disorders, or new lesions on CT or MRI scans [5].

Early diagnosis and treatment of a concussion are crucial for positive outcomes, as misdiagnosis or delayed treatment can result in serious complications, such as an increased risk of disability and mortality [2]. This is particularly relevant in the case of repeated concussion insults, as commonly seen in football. Therefore, it is important to follow current guidelines based on the best available evidence to ensure the appropriate treatment of athletes and to facilitate a safe return to athletics and daily activities. This literature review aims to provide insight into the proper evaluation of concussions and potential treatment options, as there is no definitive treatment approach.

## Review

Study design

A comprehensive literature search from April 2012 to April 2022 using PubMed, Embase, and Medline databases was performed. The inclusion criteria sought studies that were peer-reviewed, written in English, and performed on human subjects. The exclusion criteria involved non-English studies, animal studies, and literature reviews. The key terms used in the search included "concussion," "mTBI," "effects," "recovery," "therapeutics," “OMM," "osteopathic manipulative medicine," "athletes," "sports," “return to play,” and "treatment." The initial search resulted in 163 articles; a second-tier review focused on articles involving athletes with diagnosed concussions and treatment approaches, resulting in the final 24 articles to be included in the literature review. In 2024, it was noted that the Amsterdam 2022 International Consensus Statement on Concussion in Sport was released and thus was included in this review to explore the recent developments in concussions.

Review

Signs and Symptoms of Concussions

A concussion is caused by a direct blow to the head, face, or neck that can cause temporary neurological impairments, which typically resolve rapidly. However, some symptoms can persist for a longer time or emerge over time. These symptoms that may emerge include emotional or personality changes, cognitive impairments, difficulty sleeping, and physical symptoms such as tinnitus, vision changes, and dizziness. If symptoms seem to persist or worsen, further assessment and treatment may be necessary [14].

The post-concussion symptom scale (PCSS) is a self-reported measure involving the Likert scale [15]. The Likert scale consists of the 22 most common symptoms associated with a concussion [15]. This comprehensive approach is crucial given the variability of concussion symptoms in an individual. The symptoms of a concussion typically include headache, nausea, vomiting, balance impairment, dizziness, fatigue, trouble falling asleep, increased or decreased sleep, mood changes, sensitivity to light and/or noise, numbness, tingling, difficulty concentrating, difficulty with memory, and visual problems [15]. It is important for clinicians to use a multifaceted approach when evaluating and managing head injuries because concussion injuries can affect different neural networks and the combination of symptoms can present uniquely in each patient [15]. It is important to recognize the signs and symptoms of concussions in different age categories as younger athletes can demonstrate worse post-concussion neurocognitive performance than older athletes [15].

Physical Assessment

A comprehensive evaluation of a concussion cannot be achieved through a singular assessment method. A combination of tools must be used to examine a concussion [15]. The general acute evaluation of a patient with a concussion involves mental status and neurological testing [16]. Additionally, orthostatic vital signs, cranial nerves, vestibular, ophthalmologic, and cervical systems must be assessed on initial physical evaluation. The Brief Buffalo Concussion Physical Exam (BCPE) tests these aspects and can be easily administered during subsequent follow-up appointments to monitor treatment efficacy [17].

Head injuries commonly present with dizziness or orthostatic hypotension, and therefore, vital signs are imperative to monitor. Patients with hypotension or dizziness upon standing may have a central vestibular injury, while patients with those symptoms while supine may have a peripheral vestibular injury [17]. Cranial nerves must also be assessed in patients with concussions. Abnormalities in any cranial nerve may suggest a brainstem injury, which will warrant further testing [17]. Additionally, a fundoscopy must be performed to test ophthalmologic irregularities. Patients with concussions may present with repetitive saccades, abnormal smooth pursuits, and abnormal accommodation [17]. The cervical spine must also be assessed to determine injury due to physical trauma. The vestibular system requires thorough assessment by trained physical therapists; balance impairment is often tested using the Balance Error Scoring System (BESS) [17].

The most crucial consideration following a sports-related concussion is determining the appropriate timing for return to play. Exercise tolerance must be examined with an aerobic treadmill test such as the Buffalo Concussion Treadmill Test (BCTT). The BCTT has been shown to be safe even if performed one week post-concussion. On the other hand, activities such as weightlifting or sprinting can cause a rapid increase in heart rate, which is not recommended when assessing symptoms in a patient [17].

Rest and Hydration

The fundamental approach to treating concussions requires ensuring adequate rather than excessive cognitive and physical rest [5]. Although the available evidence is limited, it is recommended to sustain a period of rest during the acute symptomatic phase (24-48 hours) following a concussion. This period of rest involves limiting cognitive, physical, and social activities that may include reducing school attendance, academic work, electronics usage, and sports-related activity. Rest is commonly thought to potentially reduce symptoms and expedite recovery time, while also decreasing the risk of repeat concussion and the rare, but potentially fatal, second impact syndrome [5]. Second impact syndrome is a condition that can occur when an individual experiences a second concussion before the symptoms of the first concussion have resolved. It can cause rapid and severe swelling of the brain, leading to increased intracranial pressure, brain herniation, and death. However, the study conducted by Buckley et al. determined that individuals who did not rest following a concussion had a faster recovery compared to those who rested [5].

JVC Collar

In football and many other sports, helmets are used to protect the skull from fractures, but they do not protect the brain from injuries. To reduce the acceleration and deceleration force known to contribute to concussive insult, helmets and neck collars are current areas of research. There is potential for the use of a neck collar that applies bilateral jugular vein compression, which can induce venous engorgement and reduce energy absorption during head impacts [6]. The purpose of venous engorgement is to facilitate a tighter fit of the brain within the cranium [6]. Ultrasound was used to confirm that the jugular vein increased in size after the collar was placed, and the head impact was the same between the control and collar groups. The use of the collar prevented diffusion changes in the brain after repetitive head impacts during a football season [6]. This finding is important for the future of sports as it shows that there may be use for the collar to prevent brain injuries. In athletes with concussions, MRI scans have shown decreased fractional anisotropy (FA) and increased mean diffusivity (MD) ratio, which exemplifies damage to the myelin sheath and axon membrane due to swelling or inflammation [6]. There was no change seen in the group with the collar, which is promising in potentially avoiding such internal brain damage in the future with the use of a collar. Although this study took into consideration similar impact, it is important to consider outside stressors such as other present traumas, genetics, age, and gender [6].

Osteopathic Manipulative Medicine Treatment (OMT)

Some providers have attempted to manage headaches associated with mTBI using OMM. OMM or OMT is a therapeutic treatment approach with a focus on the musculoskeletal system. It incorporates a comprehensive physical exam aimed at evaluating somatic dysfunctions and disruptions or deviations in bodily function. Through OMM, dysfunctions within the musculoskeletal system and its interconnected fascia are purported to be alleviated using specific techniques [8,18]. While evidence is limited, a recent study suggests a reduction in post-concussive symptoms with the use of OMM [7]. The focus of the study was the improvement of post-mTBI headaches. Individuals with concussion-related headache symptoms for at least three months were randomly assigned to a control group receiving no OMT treatment and an OMT treatment group. In this study, suboccipital release, counterstrain, muscle energy, and myofascial release were selected due to their widespread use and minimal risk of adverse effects [7]. After treatment, pain intensity was assessed immediately post-treatment using the Visual Analog Scale (VAS) and a Headache Impact Test (HIT-6). Within the treatment group, the average difference between baseline VAS scores and immediately post-treatment was statistically significant, and no adverse effects were noted [7]. While there was an improvement in HIT-6 scores after OMT, no statistically significant difference was found between scores. This study demonstrated an immediate improvement in VAS scores after OMT, but further evidence for consistent and long-term improvement and generalization is necessary [7].

Vestibular dysfunctions, including dizziness and eye-head coordination impairment, are some areas wherein authors have attempted to study the impact of OMT [8]. In the study conducted by Chappell et al., the severity of the symptoms was measured using the Sports Concussion Assessment Tool-2 (SCAT2) scale, with zero defined as no symptoms and 6 being severe symptoms [7]. The SCAT2 is a standardized tool used to assess athletes who may have sustained a concussion, measuring cognitive, physical, and neurological functions. Versions of this tool are used to help medical professionals determine the severity of concussion and monitor recovery progress [8]. During evaluation, the choice of OMT techniques to treat somatic dysfunction in the patient was left to the discretion of the treating physician and involved both direct and indirect techniques [6]. After administering OMT to each patient, another SCAT2 assessment was completed to compare pre-treatment and post-treatment scores [8]. The study concluded that after OMT treatment, there was an improvement in symptoms of headache, pressure, balance impairments, noise sensitivity, fogginess, difficulty concentrating, fatigue, irritability, and sadness [7]. A few limitations of this study include variability in treatments selected by different treating clinicians, lack of a control group and randomization, sample size, and the use of an outdated version of the SCAT tool [7]. These factors limit our understanding of the efficacy of this treatment approach.

OMT has also been studied for its use in lymphatic drainage and circulation with inconclusive findings [8]. Ultimately, these studies assessing the efficacy of OMT within this realm are limited by variability in treatments selected by differing clinicians, lack of a control group and randomization, low sample size, and the use of an outdated version of the SCAT tool [7]. Thus, variability in methodological quality and lack of sufficient support for short- and long-term benefits limit our understanding of the efficacy of this treatment approach.

Herbal Supplement and Pharmacotherapy

After an mTBI, the brain restores cells and neuronal connections that have been damaged. Meanwhile, reactive oxygen species and antioxidant balances are disturbed and can lead to oxidative stress [9]. It has been demonstrated that after an mTBI, antioxidant levels are low so supplementing the body with antioxidants may aid in recovery [9]. The herbal supplement MLC901 (NeuroAiD II) is a traditional Chinese medicine that is purported to restore neuronal damage from antioxidants, stimulate axons and neurons, and cause cell proliferation, which can lead to potential improvement in cognitive function and decreased neurodegeneration [9]. Cognitive function is most affected after a concussion; treatment with MLC901 showed improvement in complex attention and executive functioning as well as cognitive function over a period of six months [9].

Methylphenidate is a first-line treatment for impairment in executive brain function and information processing speed, with usefulness when cognitive changes are present [10]. One of the most common symptoms of mTBI is cognitive changes; therefore, methylphenidate is a potential adjunctive therapy. In the study performed by McAllister et al., the efficacy of methylphenidate and galantamine on the treatment of post-concussion syndrome was investigated, and it was shown that methylphenidate improves cognitive complaints, attention, speed of information processing, and depressive symptoms, while galantamine improves episodic memory and depressive symptoms [10].

Hyperbaric Oxygen Therapy (HBOT) and Blood Flow Restriction

Persistent post-concussion syndrome (PPCS), which can result from an mTBI, may lead to long-term complications. Meanwhile, consensus data regarding the most effective treatment approaches remain limited. HBOT has been purported to improve cognitive functioning and relieve symptoms in patients exposed to brain trauma [11]. In one study, at a frequency of five days a week, over a 40-day period, 150 kPa HBOT treatments were provided to patients for one hour. After treatment, the researchers found a significant improvement in symptomatology including memory, speed of information processing, and depression in patients [11]. The only symptom among patients that worsened was fatigue, which is hypothesized to be a sign of oxidative stress, as previously reported [11]. This study suggests that HBOT may serve as a potentially beneficial treatment for mTBI PPCS, but further study is needed.

Exercise can treat sports-related concussions and concussion symptoms effectively [12,19]. Another study aimed to evaluate the effectiveness of moderate-intensity interval training with blood flow restriction and body cooling among patients with symptoms of concussion for less than one year [20]. The researcher found that there was less fluctuation in symptom severity among patients who underwent moderate-intensity interval training, which was defined as 65% predicted maximal heart rate. In addition, more stable recovery was observed in patients who exercised using blood flow restriction and body cooling [20]. Overall, the researchers concluded that moderate-intensity interval training with blood flow restriction and body cooling reduced concussive symptoms in study participants [20]. This is an important finding as it can be useful to aid recovery in athletes. Many athletes are eager to get back into sports; offering them moderate-intensity interval training in their recovery plan can keep them motivated and in shape as they work toward recovery.

Aerobic Exercise and Vestibular Rehabilitation Therapy (VRT) in Concussion Recovery

Aerobic exercise has also been tested to determine its efficacy in treating adolescents with sports-related concussions and concussion symptoms. Male and female adolescents who suffered a concussion within the last 10 days performed aerobic exercises and showed improvement in symptoms within four weeks and had a 48% decreased risk of long-term post-concussive symptoms [12]. This finding elucidated the importance of early treatment of concussion with aerobic exercise, contrary to the traditional approach of prolonged rest. As frontline providers in treating concussions, these findings suggest that physicians should consider prescribing early light aerobic physical activity to adolescents as a treatment to hasten recovery and potentially reduce the risk of developing post-concussive symptoms [12]. Individuals with chronic symptoms demonstrated a faster rate of improvement with the use of aerobic exercise. The level of intensity was determined by the use of an Activity Intensity Scale (AIS) to help categorize activity levels after injury [14]. The self-reported activity was dictated within patient records by the clinician performing the evaluation. The difference in activity level was determined by a single rater at each evaluation interval [14]. As it relates to the dosage of aerobic exercise, Majerske et al. concluded that an intermediate level of activity reduces symptoms the most. In this study, intermediate activity included activity at school and light activity at home, such as mowing the lawn or jogging [15]. Like the use of moderate-intensity interval training as a treatment, the use of aerobic exercise as a treatment for concussion and reducing post-concussive symptoms will allow athletes to be able to exercise to maintain a level of conditioning as well as help with recovery [21]. For concussion management, aerobic conditioning can be managed during physical therapy, preferably by a physical therapist with experience or board specialization in orthopedics, sport, neurology, and/or cardiovascular and pulmonary physical therapy.

Dizziness and balance dysfunction have been reported in up to 80% of cases of concussion within the first few days of injury [22]. Physical therapists manage patients with mTBIs through vestibular, balance, and neuro-musculo-skeletal rehabilitation [12]. Concussions have been shown to be associated with neuron impairment within the vestibulospinal tracts, leading to dysfunctions in balance and gaze resulting in symptoms of dizziness. The dysfunction in balance and gaze as well as symptoms of dizziness can persist and cause long-term impairments to an individual’s personal life. In a study performed by Kleffelgaard et al., VRT performed by physical therapists included Brandt-Daroff exercises for vertigo and gait stabilization. Home exercises were also encouraged and included walking and biking [13]. The treatment was tailored to each participant based on their symptoms. The study showed that in addition to the usual treatment for TBI, group-based vestibular rehabilitation intervention led to an improvement in dizziness and mobility symptoms in the treatment group [13]. Meanwhile, by the second post-study follow-up, both the treatment and control groups were able to achieve the same level of improvement. Vestibular rehabilitation can potentially speed up the recovery process following a concussion, in terms of dizziness-related dysfunctions [13]. In addition, it has been shown that starting VRT within the first 30 days after a concussion leads to an earlier return to play and resolution of symptoms [13]. Further research is warranted to determine the long-term effects of individual- versus group-based VRT in the resolution of symptoms. Table 1 provides a summary of the treatment methods discussed.

**Table 1:** A summary of the treatment options described within the review article, including rest and hydration, JVC collar, OMT, herbal supplement, pharmacotherapy, hyperbaric oxygen therapy, blood flow restriction, aerobic exercise, and VRT.

Treatment	Summary of Treatment
Rest and hydration	Rest for 24-48 hours after injury [5].
Jugular vein compression (JVC) collar	Bilateral jugular vein compression reduces energy absorption by facilitating a tighter fit of the brain within the cranium [6].
Osteopathic manipulative medicine treatment (OMT)	Alleviates musculoskeletal and fascia tension. The treatments included suboccipital release, counterstrain, muscle energy, myofascial release, and lymphatic system drainage [8].
Herbal supplement and pharmacotherapy	Low antioxidant levels following mTBI can be treated with an herbal supplement, MLC901 (NeuroAiD II), which is postulated to restore neuronal damage, stimulate axons and neurons, and cause cell proliferation [9]. Methylphenidate is a first-line treatment for executive brain function impairment and has been shown to improve cognition, attention, speed of information processing, and depressive symptoms [10]. Galantamine can be used in conjunction with methylphenidate to improve depressive symptoms and episodic memory [10].
Hyperbaric oxygen therapy, blood flow restriction	HBOT to treat persistent post-concussion syndrome by improving cognitive function, memory, depression, and information processing speed, but some patients experienced worsening fatigue. Thus, further study is needed [11]. Moderate-intensity interval training with blood flow restriction and body cooling can be used to aid athletes in recovery as it may show less fluctuation in symptom severity and a more stable recovery [20].
Aerobic exercise	The level of intensity of aerobic exercise can be determined using an Activity Intensity Scale [19]. The performance of light aerobic physical exercises within 10 days post-concussion showed improvement in patient symptoms within four weeks and decreased risk of long-term symptoms [12].
Vestibular rehabilitation therapy (VRT)	Vestibular rehabilitation therapy has shown improvement in dizziness and mobility symptoms after a concussion if initiated within 30 days of the concussion [13]. VRT includes, but is not limited to, exercises for vertigo and gait stabilization, such as walking and biking, but must be tailored to each patient [13].

Return to Learn and Play Considerations

Studies have concluded that the neurochemical changes in the brain resulting from head impacts can lead to a decline in cognitive functions [23]. Due to reduced cognitive functions, it is important to determine what the correct timeframe is for athletes to return to learn and play. Concussion-induced dysfunction is likely tied to decreased cerebral blood flow (CBF) and disrupted neurotransmission from injuries to axons, which can impair memory and attention [23]. Thus, there is a correlation between an increased number of concussions experienced and cognitive decline [23]. Another important consideration when deciding readiness for return to play is reaction time in relation to short-term memory. It is shown that a history of multiple concussions will impair short-term memory formation [23]. Due to this relationship between concussions and memory formation, it is important to consider the history of concussions in an athlete to determine when it is appropriate for them to return to learn.

In addition, an effective rehabilitation strategy for athletes who have experienced a concussion must be established to ensure a safe return to play. Currently, there is an accepted graduated stepwise recovery strategy that begins with a short period of rest. The athlete should have approximately 24-48 hours of rest (Table 1) [24]. After this, it is recommended that the patients begin limited activity while remaining below a cognitive and physical exacerbation threshold. If concussion-related symptoms resolve, the athlete can continue to proceed to light aerobic exercise (Table 1) [24]. After performing light aerobic exercise without recurrence of symptoms, the patient can perform sport-specific exercises, non-contact training drills, full-contact practice, and finally return to sport and normal gameplay. These steps of increasing intensity should be performed at least 24 hours apart to allow the athlete a minimum of one week to fully recover. This timeframe will differ based on the age, level of sport, and history of the patient [24]. However, this process for rehabilitation should be used for the management of athletes regardless of age or level of the sport.

Recent Developments in mTBI Management

The Amsterdam 2022 International Consensus Statement on Concussion in Sport provides a consensus methodology to update recommendations for sport-related concussions. The statement offers practice recommendations for healthcare professionals caring for athletes [25].

The key policy changes and findings are summarized in Table 2 [25].

**Table 2:** The Amsterdam 2022 International Consensus Statement on Concussion in Sport key findings.

Categories of the Changes	Key Findings
Youth ice hockey	Body checking is no longer allowed, resulting in a 58% reduction in concussion rates. Suggests extending this policy to most levels of adolescent hockey players.
Ice hockey in general	The use of mouthguards led to a 28% reduction in concussion rates across all age groups.
American football	Imposing limitations in practices to restrict collision time resulted in a 64% reduction in practice-related concussions across all age groups.
Rugby	On-field neuromuscular training (NMT) warm-up programs at least three times a week showed lower rates of concussion across all age groups.
Immediate removal for suspicion of concussion	Players showing signs like loss of consciousness, seizures, ataxia, poor balance, confusion, or amnesia should be removed immediately. Return to play is only allowed after medical clearance if such signs are present.
Diagnostic tests and evaluations	In addition to the SCAT, new tests include a 12-word list (5-word list prior) and timed dual gait tasks. The SCAT6 should be used when a player is removed from play during the game, at halftime or at the end of the game. It should be followed up 24-48 hours after the injury. Measurement of blood pressure and heart rate, a full neurological examination, and an assessment of cervical spine tenderness should be included in the initial evaluation. Symptoms that the parents and/or child report should also be documented.
Post-concussion management	Persistent symptoms, like dizziness, neck pain, or headaches beyond 10 days, require cervicovestibular rehabilitation. After four weeks of persistent symptoms, active rehabilitation and collaborative care may be considered.
Rest recommendations	Relative rest is recommended for 48 hours after a concussion, with a gradual increase in exercise intensity based on symptoms. Relative rest is defined as completing daily living tasks with mild activity. Prior to this Statement, rest was recommended for 24-48 hours with absolutely no return to daily living or screen time. Reduced screen use within 48 hours may be effective in alleviating neurological symptoms like headaches. It is not beneficial after the 48-hour mark to restrict screen time.
Return to learning	In children, adolescents, and young adults, modifications are important for school attendance, increased rest breaks, limiting electronic devices, and extra time and/or delaying assignments and exams. Contact sports with potential head collisions and falls must be avoided until full recovery.
Return to sport guidelines	Relatively the same: rest for 24-48 hours followed by a stepwise approach of return to activity. The stepwise approach begins with rest and then goes to light activity, moderate activity, sport-specific activity, non-contact drills, and full-contact practice. Medical clearance is required before full-contact practice.

Discussion

The Consensus statement, published in 2023, provided updated information on concussion management (Table 2). While many of the protocols remain consistent, they now emphasize more individualized treatment plans based on the signs and symptoms that the player is experiencing.

Concussions are diagnosed clinically based on a medical provider’s physical examination. There is no specific imaging or lab test that can diagnose a concussion. Additionally, the duration and type of symptoms can vary depending on the patient. Concussions commonly present with symptoms such as nausea, headache, vomiting, and dizziness [2]. Furthermore, there is no definitive treatment for concussions since symptoms can vary so greatly and treatment regimen is heavily dependent on that variance. Currently, medical providers recommend a stepwise approach to full physical activity beginning with a period of 24-48 hours of rest (Table 1). Proper rest and recovery limit physical activities, which can lead to the reduction of symptoms [5]. Recent studies have elucidated the effects of various treatments in addition to rest and hydration including the use of a JVC collar, OMM, pharmacotherapies, HBOT, aerobic exercise, and physical therapy to improve concussion symptoms (Table 1).

The JVC collar causes bilateral jugular vein compression leading to venous engorgement and reduction in energy absorption when head impact occurs [6]. This is purported to allow a tighter fit of the brain into the cranium, with the hope of mitigating further brain injuries. OMM focuses on manual musculoskeletal treatments; suboccipital release, counterstrain, muscle energy, and myofascial release were evaluated and may help in treating dizziness and coordination impairment [7]. Lymphatic treatment, which enhances the circulation of the lymph system, aims to reduce symptom duration and severity by helping to decrease inflammation from injury [8]. Additionally, herbal supplements and pharmacotherapy are potential adjunct therapies. MLC901 (NeuroAiD II) is a traditional Chinese medicine theorized to restore neuronal damage by allowing cell proliferation, further improving cognitive function [9]. From the pharmacological standpoint, methylphenidate improves executive brain function and processing speed, which can improve the cognitive disturbance that results from a concussion [10]. Galantamine is another pharmacological therapy that can improve depressive symptoms and memory when used in conjunction with methylphenidate [10].

When PPCS arises, long-term complications are more likely. There are limited data on the treatment of PPCS. The efficacy of HBOT is being assessed as a means to improve memory, speed of information processing, and overall cognitive function after a concussion [11]. Moderate-intensity interval aerobic training can help improve symptoms as well as decrease long-term concussion symptoms [12]. The AIS is used to determine the level of aerobic intensity for each individual athlete [19]. Light activity within 10 days of a concussion can improve patients' symptoms within four weeks and decrease the chance of long-term sequelae [12]. Lastly, VRT helps to improve symptoms of vertigo and changes in gait and mobility, among other vestibular system-based impairments, particularly if completed within 30 days of a concussion [13].

The Amsterdam 2022 International Consensus Statement on Concussion in Sport determined new updated guidelines in the approach to concussions. The guidelines have been relatively consistent in the treatment approach; however, there were a few recommendations based on recent studies: players should be immediately removed if neurological signs such as ataxia, loss of consciousness, and amnesia arise; body checking in youth ice hockey is no longer allowed; the use of mouthguards in ice hockey decreases the concussion risk; and the use of neuromuscular training in warm-ups of rugby decreases the rates of concussions [25]. The SCAT6 tool is recommended for use when a player is removed during a game and followed up 24-48 hours after injury [25]. The rest recommendations have been modified to include relative rest for 48 hours, which consists of completing daily tasks and mild activity, but no exertion [25]. Screen time may be useful when eliminated for 48 hours after sustaining a concussion, but it is not necessary [25].

Limitations

There were a few notable limitations to the studies reviewed. First, the implementation of uniform study designs is needed to yield results that are not only more precise but also have greater applicability to clinical practice. This would facilitate replicability and enhance the validity of the findings. Second, a large, diverse sample size that considers patient gender and age is important for future research. Additionally, there are many factors that impact recovery from concussions, and it is important to consider extrinsic factors such as genetics, stressors, and the effects of concurrent treatments. Lastly, a notable limitation in many studies is the lack of a long-term follow-up with participants, resulting in a lack of data on the long-term efficacy and outcomes of the therapy.

## Conclusions

Concussions are a type of mild traumatic brain injury that can lead to severe long-term complications. They are heavily prevalent in high-impact sports such as football and boxing. Symptoms of concussion include dizziness, nausea, vomiting, headaches, and photosensitivity, which are common in many other injuries and illnesses. This overlap makes diagnosis challenging, as expert consensus currently points to clinical signs and symptoms for diagnosis. The diagnosis of concussion is based on physical evaluation, and early diagnosis and management are crucial for recovery. A physical exam must include an evaluation of orthostatic vital signs, cranial nerves, vestibular, ophthalmologic, and cervical systems, as well as mental status and neurological testing. Currently, there are many treatments that help with concussion management, such as relative rest, physical therapy (typically vestibular-based rehabilitation), graded aerobic exercise, and optometry. Many of these therapies should be used in conjunction with each other to achieve faster and more effective resolution of symptoms. For athletes, return to play time is crucial to prevent the worsening of physical and cognitive symptoms. Once a concussion is diagnosed, it is important to be treated immediately and expertly through an interdisciplinary team approach to afford athletes the best chance at full recovery as they return to athletics and education. The Amsterdam 2022 International Consensus Statement on Concussion in Sport provided an update on recommendations for sport-related concussions.
